# Machine learning enables early risk stratification of hymenopteran stings: evidence from a tropical multicenter cohort

**DOI:** 10.3389/fpubh.2025.1664606

**Published:** 2025-10-28

**Authors:** Feng Han, Yuanshui Liu, Huamei Li, Xiaofang Chen, Liqiu Liang, Dongchuan Xu, Lijiao Ye, Yanhong Ouyang, Ping He, Wang Liao

**Affiliations:** ^1^Department of Emergency Medicine, Hainan General Hospital and Hainan Affiliated Hospital of Hainan Medical University, Haikou, China; ^2^Department of Ultrasound, Hainan General Hospital, Hainan General Hospital and Hainan Affiliated Hospital of Hainan Medical University, Haikou, China; ^3^Biomedical Statistics Office, Hainan General Hospital and Hainan Affiliated Hospital of Hainan Medical University, Haikou, China; ^4^Department of Cardiology, Hainan General Hospital and Hainan Affiliated Hospital of Hainan Medical University, Haikou, China; ^5^Hainan Clinical Research Center for Cardiovascular Disease, Haikou, China

**Keywords:** hymenopteran stings, machine learning, risk stratification, emergency triage, model interpretability, epidemiology

## Abstract

**Background:**

Hymenopteran stings (from bees, wasps, and hornets) can trigger severe systemic reactions, especially in tropical regions, risking patient safety and emergency care efficiency. Accurate early risk stratification is essential to guide timely intervention.

**Objective:**

To develop and validate an interpretable machine learning model for early prediction of severe outcomes following hymenopteran stings.

**Methods:**

We retrospectively analyzed 942 cases from a multicenter cohort in Hainan Province, China. Questionnaires with >20% missing data were excluded. Mean substitution was applied for primary missing data imputation, with multiple imputation by chained equations (MICE) used for sensitivity analysis. Seven supervised classifiers were trained using five-fold cross-validation; class imbalance was addressed using the adaptive synthetic sampling (ADASYN) algorithm. Model performance was evaluated via area under the receiver operating characteristic curve (AUC), recall, and precision, and feature importance was interpreted using Shapley additive explanations (SHAP) values.

**Results:**

Among 942 patients, 8.7% developed severe systemic complications. The distribution by species was: wasps (25.5%), honey bees (8.9%), and unknown species (65.6%). The optimal Extra Trees model achieved an AUC of 0.982, recall of 0.956, and precision of 0.926 in the held-out validation set. Key predictors included hypotension, dyspnea, altered mental status, elevated leukocyte counts, and abnormal creatinine levels. A web-based risk calculator was deployed for bedside application. Given the small number of high-risk cases, these high AUC values may overestimate real-world performance and require external validation.

**Conclusion:**

We developed an interpretable, deployable tool for early triage of hymenopteran sting patients in tropical settings. Emergency integration may improve clinical decisions and outcomes.

## 1 Introduction

Hymenopteran stings (from bees, wasps, and hornets) are a common cause of outdoor injuries worldwide, particularly prevalent in tropical and subtropical regions (1), Hymenopteran venoms contain a variety of active components, including phospholipase A, hyaluronidase, histamine, and hemolytic toxins, which can trigger local inflammatory reactions, In severe cases, it may lead to anaphylactic shock, hemolysis, renal dysfunction, rhabdomyolysis, and even multiple organ dysfunction syndrome (MODS) (2). Although most patients recover well, a subset may deteriorate rapidly within a short period, resulting in serious adverse outcomes or death.

In the United States, an estimated 30 to 50 deaths due to hymenopteran stings occur annually. In developing countries, limited access to healthcare and inadequate public knowledge of emergency response contribute to a higher fatality rate. In China, seasonal outbreaks of hymenopteran sting incidents have been reported in provinces such as Shaanxi, Yunnan, and Hainan, occasionally causing multiple deaths (3). In recent years, climate change and the expansion of human activity have led to a broader distribution of hymenopteran populations and a rising trend in sting cases, posing increasing public health concerns.

Clinical outcomes following hymenopteran stings vary greatly and are influenced by multiple factors such as sting site, number of stings, history of allergy, underlying diseases, and delays in seeking medical attention. However, systematic studies evaluating and quantifying the combined effect of these variables are lacking. Current risk stratification relies on empirical judgment, which is often insufficient in emergencies. An interpretable risk assessment tool is urgently needed to assist frontline emergency physicians in identifying high-risk patients at the time of initial consultation and guiding treatment strategies and resource allocation.

Recent studies have emphasized the growing burden of Hymenoptera stings globally, with a marked seasonal surge in tropical and subtropical regions, especially during warmer months when bee and wasp activity peaks (1). In countries like Thailand and Brazil, regional apicultural density and environmental exposure patterns have been linked to increased sting incidence and poor clinical outcomes (4). Climate change and urbanization have further altered hymenopteran distribution patterns, with implications for public health emergency preparedness (5).

The pathophysiology of hymenopteran venom involves both direct cytotoxicity and immunologic hypersensitivity. Toxins such as phospholipase A2, melittin, and hyaluronidase disrupt endothelial integrity, provoke mast cell degranulation, and can lead to multi-organ failure in severe cases (6). These mechanisms explain the clinical correlation observed between venom load and elevations in leukocyte count and serum creatinine—key features explored in our model. Recent advances in understanding hymenopteran venom composition and its clinical implications have highlighted the need for species-specific risk assessment tools (7).

The integration of artificial intelligence in emergency medicine risk stratification represents a paradigm shift from empirical clinical judgment to evidence-based, data-driven decision making (8). Our SHAP-enhanced approach addresses the critical “black box” limitation of machine learning models in healthcare, providing clinicians with interpretable explanations for each prediction. This transparency is essential for clinical adoption and regulatory compliance, as it allows physicians to understand and validate the model’s reasoning process (9). Recent studies have demonstrated that interpretable AI models significantly improve clinician trust and decision-making accuracy in emergency settings (8).

Despite mounting data, most existing triage protocols for hymenopteran stings remain empirically driven. Scoring systems such as Sequential Organ Failure Assessment (SOFA), quick Sequential Organ Failure Assessment (qSOFA), and National Early Warning Score (NEWS) have demonstrated moderate performance in sepsis-related syndromes but are ill-adapted to envenomation scenarios, which often present with rapid systemic deterioration but distinct biomarkers (10). Recent advances in machine learning and artificial intelligence have shown promise in emergency medicine risk stratification, with applications ranging from sepsis prediction to allergic reaction severity assessment (11).

To our knowledge, no previous studies have systematically applied interpretable machine learning models to stratify hymenopteran sting severity at initial presentation. Our study addresses this critical gap using multicenter real-world data and proposes an accessible, validated digital tool for clinical deployment.

Building upon recent progress in interpretable machine learning models for medical risk prediction, our study represents the first to systematically apply such methods to hymenopteran sting patients. Leveraging multicenter data from Hainan Province, we developed a robust, explainable prediction model and an accessible web-based tool to support early triage and resource allocation.

## 2 Methods

### 2.1 Study population

This retrospective study included patients who suffered hymenopteran stings and received treatment at five randomly selected secondary or higher-level medical institutions in Hainan Province between January 1, 2019 and December 31, 2021. Inclusion criteria were confirmed history of hymenopteran sting exposure and complete clinical data, exclusion criteria were absence of a confirmed clinical diagnosis or incomplete clinical records. A total of 1,102 questionnaires were collected; those with more than 20% missing data were excluded, leaving 942 valid cases for analysis. For the primary analysis, missing values were initially handled using mean substitution. We validated robustness via sensitivity analysis with MICE. The MICE-imputed dataset showed superior stability and predictive performance compared to mean substitution (Table 1), and was therefore adopted as the main analysis pipeline. Patients were classified as high risk (death, requirement for continuous renal replacement therapy, multiple organ dysfunction syndrome, or other severe complications) or low risk (favorable outcomes without significant complications, organ failure, or critical interventions).

**Table 1:** Performance comparison between mean and MICE imputation methods across models.

Model type	Imputation method	AUC	Recall	Precision
Extra Trees (Full predictors)	Mean	0.959	0.919	0.866
Extra Trees (Full predictors)	MICE	0.9971	0.9783	0.9712
XGBoost (Simplified predictors)	Mean	0.9872	0.9231	0.9132
XGBoost (Simplified predictors)	MICE	0.9971	0.9783	0.9712

Performance comparison between mean and MICE imputation strategies across two classifiers. The full Extra Trees model used all available predictors, while the simplified XGBoost model was restricted to five emergency-accessible features (GCS, under_disease, low_bp, dyspnea, rash). Performance was evaluated using area under the ROC curve (AUC), recall (sensitivity), and precision (positive predictive value). MICE-imputed models consistently outperformed their mean-imputed counterparts, particularly in recall and precision, highlighting the advantages of multiple imputation in clinical risk prediction.

### 2.2 Data collection

A standardized case report form was used to collect demographics (name, gender, age), medical history (15 chronic disease categories, presence of ≥1 recorded as positive), hymenopteran sting details (location, species, date), symptoms and signs (syncope, dyspnea, urticaria, dry mouth, cold sweat), vital signs, laboratory tests (leukocyte count, creatinine, other biochemical parameters), treatments, continuous renal replacement therapy use, hospital stay, and clinical outcomes. Definitions followed standardized criteria: syncope refers to transient loss of consciousness due to temporary cerebral hypoperfusion; dyspnea is a subjective sensation of breathing difficulty; urticaria is a localized edematous reaction from vascular hyperpermeability; dry mouth refers to reduced oral moisture; cold sweat denotes perspiration associated with fear or shock, often with cold extremities; underlying disease includes chronic conditions such as hypertension, coronary artery disease, chronic kidney disease, malignancies, immunodeficiencies, and others listed in Supplementary material S4.

### 2.3 Statistical analysis

Analyses were performed using R 4.4.1. Normally distributed continuous variables were expressed as mean ± standard deviation and compared with independent samples t-test or one-way analysis of variance (ANOVA); skewed data were expressed as median (interquartile range) and compared with Mann–Whitney U test. Categorical variables were expressed as percentages and compared with chi-square test. Multivariate logistic regression was applied to identify independent risk factors for adverse outcomes. Thresholds for leukocyte count and creatinine were determined using receiver operating characteristic (ROC)–Youden index analysis via sklearn.metrics.roc_curve (Supplementary Figures S1, S2). A two-sided *p*-value <0.05 was considered statistically significant.

### 2.4 Machine learning model development (including imputation and class balancing)

The dataset was stratified into training (70%) and testing (30%) sets, with tenfold cross-validation in training for hyperparameter tuning and overfitting control. Preprocessing within each training fold included multiple imputation by chained equations using IterativeImputer in scikit-learn (random seed 123, default max_iter = 10) to handle missing values, z-score normalization of continuous variables, and ADASYN oversampling to address the imbalance of only 23 high-risk cases. ADASYN was chosen over random oversampling for its ability to adaptively focus on difficult-to-learn minority samples, improving sensitivity to rare outcomes. Thirteen classifiers (including XGBoost, Extra Trees, and CatBoost) were compared, and the best performers integrated into a stacking ensemble. Performance metrics included ROC AUC, accuracy, recall, and precision, evaluated on the held-out testing set. Models trained on MICE-imputed data consistently outperformed those using mean imputation, with notable gains in recall and precision (Table 1).

### 2.5 Simplified model development

For rapid application in emergency settings, a simplified model was built from core variables obtainable at initial assessment. Feature selection was based on clinical accessibility and ROC–Youden index thresholds. The same preprocessing and training pipeline was applied, differing only in the feature set, and predictive performance was compared with the full model. Although slightly lower in AUC, the simplified XGBoost model retained strong discrimination while reducing required features from seven to five.

### 2.6 SHAP interpretation and model deployment

SHAP analysis quantified global feature importance. In the full model, leukocyte count, low blood pressure, and creatinine were the top contributors; in the simplified model, low blood pressure, Glasgow Coma Scale <15, and underlying disease ranked highest. The final model was deployed as an online risk calculator, accepting the simplified feature set and outputting predicted probabilities with categorical risk classification, enabling real-time clinical support in emergency settings.

## 3 Results

### 3.1 Distribution of hymenopteran sting patients by gender, age, sting site, and time from sting to hospital visit

Among the 942 patients included in the study, 572 (60.7%) were male and 370 (39.3%) were female, with a male-to-female ratio of 1.55:1. The patients ranged in age from 1 to 103 years, with a mean age of 49.31 ± 20.97 years. The largest proportion of cases (34.4%) occurred in patients aged over 60 years. A total of 59 patients (6.26%) were children under 10 years of age. Additionally, 153 patients (16.2%) had underlying diseases, and 11 patients (1.2%) presented with a Glasgow Coma Scale (GCS) score of less than 15 at admission. Wasps were the most commonly identified insect (25.5%).

Regarding sting location, the head and neck were the most frequently affected areas (67.9%). In total, 527 patients (55.9%) experienced more than 10 stings. Most cases (47.9%) occurred during the third quarter. The median time from sting to hospital visit was 2 h (interquartile range: 1–4 h), with 671 patients (71.2%) presenting within 3 h of the sting incident.

The overall incidence of adverse clinical outcomes among all patients was 2.4%. Among those with adverse outcomes, 18 patients (78.3%) were stung on the head and neck, and 8 patients (34.8%) presented to the hospital within 1 h.

As shown in Table 2, in univariate analysis, there were no statistically significant differences in the incidence of adverse outcomes based on gender, age, sting site, number of stings, hymenopteran species, or time interval between sting and hospital visit (all *p* > 0.05). However, patients with underlying diseases, hypotension, or a GCS score <15 had a significantly higher incidence of adverse outcomes (*p* < 0.05). Detailed results are shown in Table 2.

**Table 2:** Demographic and clinical distribution of 942 hymenopteran sting patients in Hainan Province by gender, age, sting site, and time from sting to hospital visit.

Variable	Low-risk group (*n* = 919)	High-risk group (*n* = 23)	Total (*n* = 942)	χ^2^/Z value	*p*-value
Gender					
Male	560 (60.9)	12 (52.2)	572 (60.7)	0.722	0.395
Female	359 (39.1)	11 (47.8)	370 (39.3)		
Age (years)					
0–9	58 (6.3)	1 (4.3)	59 (6.3)	1.545	0.956
10–19	36 (3.9)	1 (4.3)	37 (3.9)		
20–29	78 (8.5)	1 (4.3)	79 (8.4)		
30–39	100 (10.9)	2 (8.7)	102 (10.8)		
40–49	136 (14.8)	4 (17.4)	140 (14.9)		
50–59	197 (21.4)	4 (17.4)	201 (21.3)		
≥60	314 (34.2)	10 (43.5)	324 (34.4)		
Underlying disease					
Yes	145 (15.8)	8 (34.8)	153 (16.2)	5.957	0.015
No	774 (84.2)	15 (65.2)	789 (83.8)		
Hymenopteran species					
Wasp	234 (25.5)	6 (26.1)	240 (25.5)	2.354	0.308
Honey bee	84 (9.1)	0 (0)	84 (8.9)		
Unknown	601 (65.4)	17 (73.9)	618 (65.6)		
Sting location					
Head and neck	622 (67.7)	18 (78.3)	640 (67.9)	1.256	0.974
Upper limb	413 (44.9)	9 (39.1)	422 (44.8)		
Hand	104 (11.3)	2 (8.7)	106 (11.3)		
Trunk	311 (33.8)	11 (47.8)	322 (34.2)		
Lower limb	318 (34.6)	10 (43.5)	328 (34.8)		
Foot	31 (3.4)	0 (0)	31 (3.3)		
Unknown	124 (13.5)	2 (8.7)	126 (13.4)		
Number of stings					
>10次	513 (55.8)	14 (60.9)	527 (55.9)	0.232	0.630
≦10次	406 (44.2)	9 (39.1)	415 (44.1)		
Time from sting to visit					
0–1 h	326 (35.5)	8 (34.8)	334 (35.5)	2.754	0.839
1–3 h	329 (35.8)	8 (34.8)	337 (35.8)		
3–6 h	118 (12.8)	2 (8.7)	120 (12.7)		
6–12 h	39 (4.2)	1 (4.3)	40 (4.3)		
12–24 h	54 (5.9)	3 (13.0)	57 (6.1)		
>24 h	33 (3.6)	1 (4.3)	34 (3.6)		
Unknown	20 (2.2)	0 (0)	20 (2.1)		
GCS					
<15	7 (0.8)	4 (17.4)	11 (1.2)	53.767	<0.001
15	912 (99.2)	19 (82.6)	931 (98.8)		
Systolic blood pressure*	130 (118, 150)	130 (115–152)	130 (115–152)	14.1643	0.6155
Diastolic blood pressure*	80 (70–88)	80 (70–88)	80 (61–87)	29.3251	0.2982
Hypotension					
Yes	25 (2.7)	12 (53.2)	37 (3.9)	145.425	0.000
No	894 (97.2)	11 (47.8)	905 (96.1)		

GCS refers to the Glasgow Coma Scale. *indicates the Mann–Whitney U test; all other comparisons were performed using the chi-square test.

### 3.2 Local and systemic manifestations in hymenopteran sting patients

Among the 942 patients, all (100%) presented to the emergency department (ED) with local skin reactions characterized by redness, swelling, or pain. A total of 47.5% of patients exhibited systemic manifestations to varying degrees, including dizziness, headache, nausea/vomiting, urticaria, syncope/coma, chills/fever, and dyspnea. In the 0–9-year age group, 44 patients (75.6%) experienced systemic symptoms. Systemic manifestations were most common in patients stung on the trunk and head, accounting for 47.4 and 45.5%, respectively. Patients who presented 3–6 h after being stung had the highest proportion of systemic symptoms (56.7%).

As shown in Table 3, there was no statistically significant association between the time from sting to hospital visit and the occurrence of systemic symptoms (*p* > 0.05). However, age was significantly associated with systemic symptoms, with higher incidence rates observed in the 0–9 and 40–49 age groups (*p* < 0.05). The presence of local symptoms was not significantly related to adverse clinical outcomes. In contrast, patients with systemic manifestations had a significantly higher incidence of adverse outcomes (*p* < 0.05). Specifically, dyspnea, cold sweat/dry mouth, syncope/coma, and generalized urticaria were significantly associated with adverse clinical outcomes (*p* < 0.05). Details are presented in Table 3.

**Table 3:** Distribution of local and systemic manifestations among hymenopteran sting patients in the tropical region of Hainan Province.

Manifestation	Low-risk group (*n* = 919)	High-risk group (*n* = 23)	Total (*n* = 942)	χ^2^ value	*p*-value
Local manifestations					
Paresthesia	44 (4.8)	1 (4.3)	45 (4.8)	1.075	0.898
Ecchymosis	64 (7.0)	1 (4.3)	65 (6.9)		
Itching	34 (3.7)	0 (0)	34 (3.6)		
Swelling	708 (77.0)	18 (78.3)	726 (77.1)		
Pain	806 (87.7)	20 (87.0)	826 (87.7)		
Systemic manifestations					
Dizziness	56 (6.1)	8 (34.8)	64 (6.8)	63.442	<0.001
Headache	43 (4.7)	2 (8.7)	45 (4.8)		
Tea-colored urine	134 (14.6)	11 (47.8)	145 (15.4)		
Nausea/Vomiting	57 (6.2)	0 (0)	57 (6.0)		
Diarrhea	12 (1.3)	0 (0)	12 (1.3)		
Syncope/Coma	7 (0.8)	4 (17.4)	11 (1.2)		
Cold sweat/Dry mouth	28 (3.0)	12 (52.7)	40 (4.3)		
Generalized urticaria	180 (19.6)	5 (21.7)	185 (19.6)		
Neurological paralysis	21 (2.3)	0 (0)	21 (2.2)		
Chills/Fever	19 (2.1)	1 (4.3)	20 (2.1)		
Shortness of breath/Dyspnea	10 (1.1)	3 (13.0)	13 (1.4)		

### 3.3 Laboratory analysis between different clinical outcome groups

As shown in Table 4, Compared with patients who had favorable clinical outcomes, those with adverse outcomes showed significantly higher levels of white blood cell count, creatinine, total bilirubin, alanine aminotransferase (ALT), aspartate aminotransferase (AST), lactate dehydrogenase (LDH), creatine kinase (CK), and activated partial thromboplastin time (APTT), along with significantly lower serum calcium levels (all *p* < 0.05). Details are provided in Table 4.

**Table 4:** Laboratory data comparison between different clinical outcome groups.

Variable	Low-risk group (*n* = 919)	High-risk group (*n* = 23)	Test statistic	*p*-value
White blood cell count (×109/L)a	13.21 ± 5.34	17.96 ± 6.10	−3.06	0.002
Serum potassium (mmol/L)a	3.73 ± 0.43	4.34 ± 1.45	−1.68	0.11
Serum calcium (mmol/L)b	2.3 (2.22, 2.41)	2.21 (2.03, 2.28)	−3.47	0.001
Creatinine (μmol/L)b	72.9 (58, 86)	98 (60.63, 205.23)	2.08	0.04
Total bilirubin (μmol/L)b	14.8 (10.07, 21.05)	24.3 (8.50, 39.18)	0.73	0.000
AST (U/L)b	33 (21.00, 55.08)	61 (41.00, 399.00)	0.152	0.000
ALT (U/L)b	29 (21.00, 44.00)	29 (17.50, 333.50)	0.133	0.000
LDH (U/L)b	239.5 (196.68, 300.9)	469 (207.3, 1839)	2.59	0.01
CK (U/L)b	230 (136.28, 397.25)	431 (125.7, 4.937)	2.25	0.03
APTT (s)b	31.25 (25.3, 44.88)	49.8 (26.9, 120.9)	2.13	0.03

APTT denotes activated partial thromboplastin time; data are presented as mean ± standard deviation (a) or median (interquartile range, IQR) (b).

Due to missing data rates exceeding 30% of total cases, the following seven laboratory indicators were excluded from subsequent modeling: total bilirubin, AST, ALT, LDH, CK, APTT, and serum calcium.

Seven potential prognostic variables were retained for machine learning model development: presence of underlying disease, Glasgow Coma Scale score <15, hypotension (defined as systolic BP < 90 mmHg or diastolic BP < 60 mmHg), dyspnea, generalized urticaria, elevated white blood cell count, and creatinine level.

To address the severe imbalance between high-risk and low-risk cases, we applied the Adaptive Synthetic Sampling (ADASYN) algorithm to the training data. ADASYN generates synthetic minority class samples in feature space, focusing more on difficult-to-learn instances, thereby improving classifier sensitivity to rare outcomes. This approach was selected over random oversampling due to its ability to adaptively shift the decision boundary and reduce bias toward the majority class.

To prevent overfitting, oversampling was confined to training folds during cross-validation. Additionally, performance was evaluated using repeated stratified k-fold cross-validation to assess robustness. While ADASYN enhances minority class representation, we acknowledge that synthetic data may not fully capture the complexity of real-world clinical cases, necessitating external validation in independent cohorts.

### 3.4 Feature selection

(1) Recursive feature elimination (RFE) and feature importance analysis

Using recursive feature elimination (RFE) and feature importance analysis based on the XGBoost model, we identified low blood pressure (low_bp), dyspnea (dysp), and Glasgow Coma Scale score (GCS) as the most critical predictors in the model. Low blood pressure consistently ranked highest across both methods, underscoring its pivotal role in predicting disease severity.

**Figure 1:**
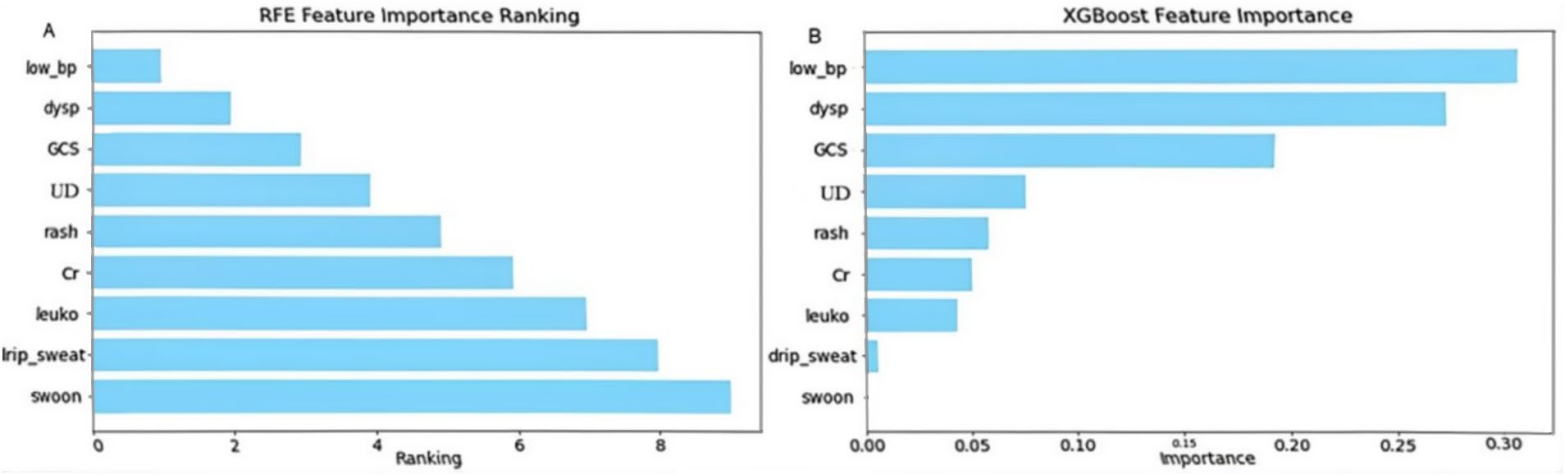
Feature importance ranking by recursive feature elimination (RFE) and XGBoost. This figure illustrates the ranking of predictor importance using **(A)** recursive feature elimination (RFE) and **(B)** the XGBoost model. leuko, Leukocytes; low_bp, Low Blood Pressure; Cr, Serum Creatinine; UD, Underlying Disease; dysp, Dyspnea; GCS, GCS<15. Axes: X-axis shows model-defined feature importance score; Y-axis shows feature names ranked by relevance.

As shown in Figure 1, Additional variables, such as underlying disease (UD), rash, serum creatinine (Cr), and leukocyte count (leuko), also contributed to model performance, although their relative importance varied. By contrast, syncope demonstrated negligible predictive relevance and was not included in the final or simplified models (Figure 1).

(2) Collinearity analysis and final selection of evaluation indicators

Collinearity analysis among the selected features revealed significant multicollinearity between low blood pressure (low_bp) and intravenous drip requirement (drip_sw). Based on the correlation strength observed in the previous step, the weaker categorical variable, drip_sw, was excluded. Consequently, the following seven features were retained for further model development: low_bp, GCS, underlying disease (under_disease), dyspnea (dysp), rash, leukocyte count (leuko), and serum creatinine (Cr).

**Figure 2:**
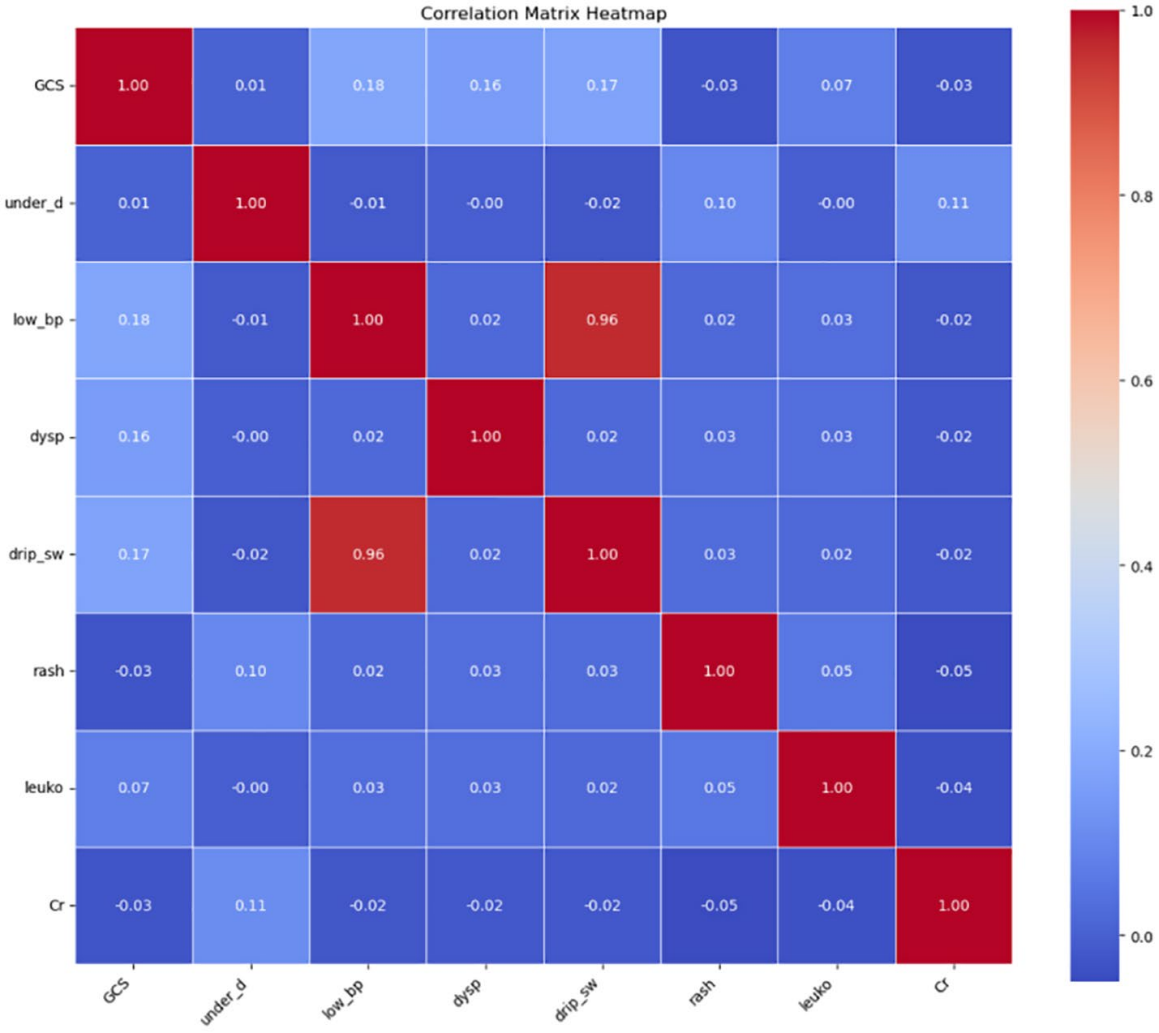
Collinearity analysis of selected features. leuko, leukocyte count; low_bp, Low Blood Pressure; Cr, Serum Creatinine; UD, Underlying Disease; dysp, Dyspnea; GCS, GCS<15.

In addition, the five clinical features that are not laboratory-dependent—low_bp, GCS, under_disease, dysp, and rash—were identified as feasible indicators for emergency triage and will be analyzed separately in the simplified risk assessment model (Figure 2; Table 5).

**Table 5:** Variance inflation factor (VIF) analysis for collinearity.

Variance inflation factor (VIF)	
Feature	VIF
GCS<15	1.066663
under_disease	1.026064
low_bp	12.883395
dysp	1.027310
drip_sw	12.847227
rash	1.021299
leuko	1.012311
Cr	1.018928

Variables with VIF > 5 were considered to exhibit multicollinearity.

### 3.5 Development and validation of an early diagnostic machine learning model

#### 3.5.1 Machine model screening

##### 3.5.1.1 Model selection using the full predictor set

We systematically evaluated multiple machine learning classifiers to identify the most suitable model for early risk prediction in severe hymenopteran sting cases. Among them, the Extra Trees classifier achieved high discrimination across evaluation metrics (AUC = 0.982, recall = 0.956, precision = 0.926), demonstrating strong capability in differentiating positive from negative cases and accurately identifying high-risk patients. Nevertheless, the limited number of high-risk cases may lead to overestimation of real-world performance, underscoring the need for validation in prospective external cohorts. XGBoost and Random Forest models performed comparably, particularly in AUC and recall, effectively capturing nonlinear feature interactions. However, their performance should likewise be interpreted with caution and confirmed in independent datasets.

Further comparative analysis showed that tree-based models—Extra Trees, Random Forest, XGBoost, CatBoost, and LightGBM—generally outperformed non-tree models. All exhibited AUCs above 0.97, demonstrating strong generalization and robust classification at varying thresholds. In contrast, the K Nearest Neighbors (KNN) model, although having a relatively high recall (0.927), suffered from lower precision (0.838), indicating a tendency for over-predicting positives. The Decision Tree model showed a relatively high recall (0.938) and AUC (0.900) but posed risks of increased false positives.

Other models such as AdaBoost, Quadratic Discriminant Analysis (QDA), Naive Bayes, Linear Discriminant Analysis (LDA), and Logistic Regression lagged behind. These models displayed lower accuracy and precision, particularly struggling with capturing the dataset’s nonlinear characteristics. Specifically, AdaBoost’s stability was inferior to other ensemble methods, while QDA and Naive Bayes failed to balance recall and precision effectively.

Overall, Extra Trees and XGBoost demonstrated comparable top-tier performance, with LightGBM achieving a respectable AUC of 0.974 but relatively weaker precision. Gradient Boosting maintained balanced performance but slightly underperformed when compared to other tree-based algorithms.

To further optimize prediction, we constructed stacked ensemble models. Ensemble A, comprising Extra Trees, XGBoost, CatBoost, and Logistic Regression as base learners and LightGBM as a meta-learner, achieved an accuracy of 0.896, AUC of 0.960, recall of 0.927, and precision of 0.872. Ensemble B, combining Extra Trees, Random Forest, and XGBoost with Logistic Regression as meta-learner, reached similar performance. Ensemble C introduced model diversity, using Extra Trees, CatBoost, and Gradient Boosting as base learners with XGBoost as the meta-learner. Ensemble D was a simplified version with Extra Trees and XGBoost.

However, none of the ensemble models outperformed the standalone Extra Trees classifier. Therefore, we ultimately selected Extra Trees as the optimal model and fine-tuned its hyperparameters to enhance prediction.

##### 3.5.1.2 Model selection based on simplified emergency department predictors

When evaluating simplified models suitable for emergency triage scenarios, single and ensemble models exhibited notable performance disparities. Among individual classifiers, XGBoost outperformed others with an AUC of 0.9397, precision of 0.8929, and recall of 0.9058, surpassing both Random Forest (AUC = 0.9387) and Extra Trees (AUC = 0.9376). Nevertheless, these simplified models performed worse than the full-feature Extra Trees model (AUC = 0.9821), suggesting that the removal of key features reduced model discrimination capacity.

Further evaluation of ensemble models revealed no improvement in performance. Ensemble A (XGBoost, Random Forest, Extra Trees, and Logistic Regression) yielded a reduced AUC (0.8709) and an alarmingly low precision (0.0968), suggesting that redundant base models introduced noise or overfitting. Ensemble B (CatBoost, LightGBM, KNN, XGBoost) showed even poorer performance (AUC = 0.8646, precision = 0.0667), highlighting the failure of non-linear stacking to capture core associations in simplified data. Ensemble C, which combined top full-feature models with a neural network, achieved the highest AUC among ensembles (0.8740) but had imbalanced recall (0.7143) and precision (0.1429), further confirming the limitations of complex integration under feature-reduced conditions.

**Figure 3:**
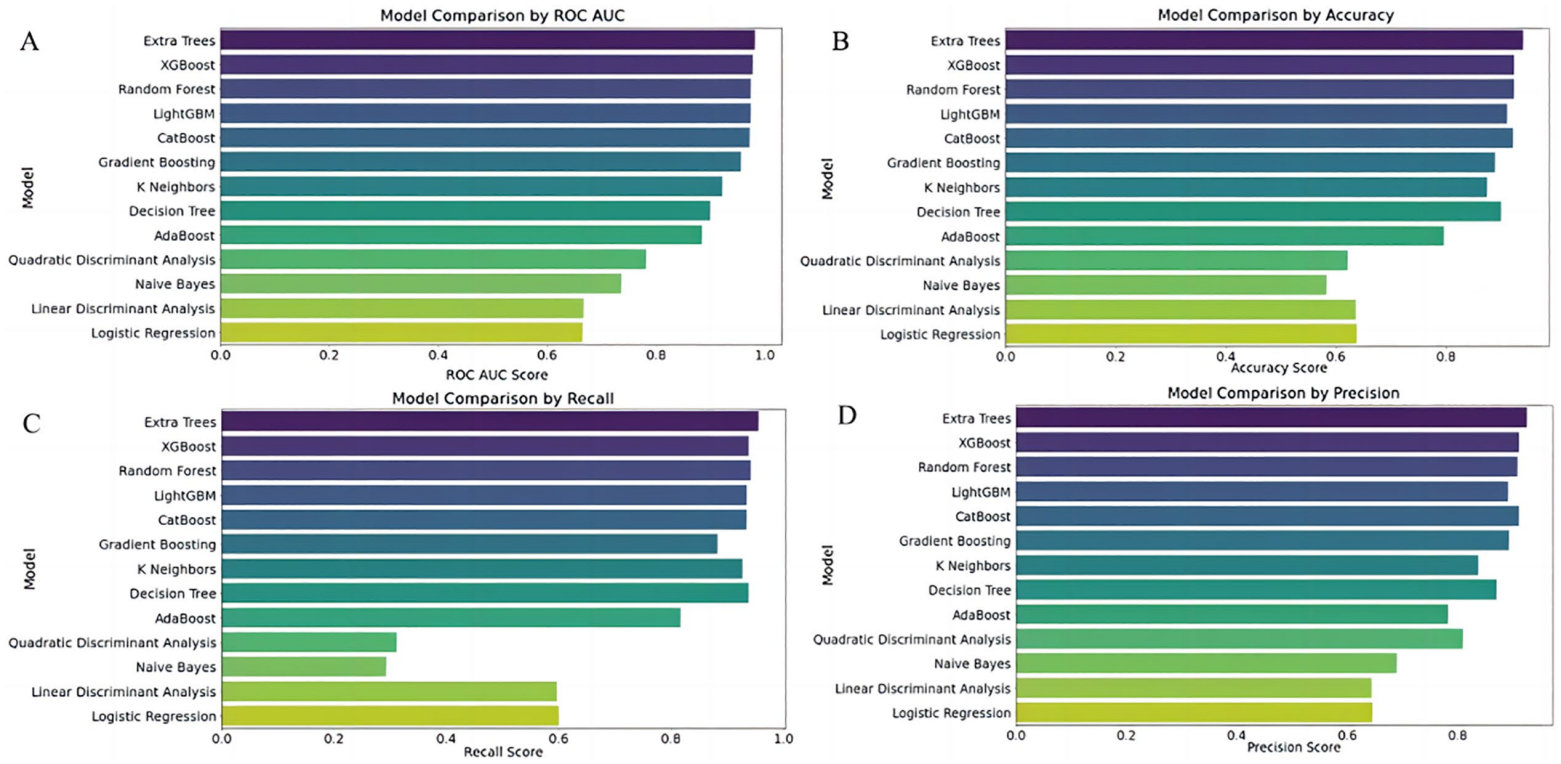
Comparative performance of 13 classifiers using full predictor set. Subfigures **(A–D)** Compare the performance of 13 classification models using four evaluation metrics: **(A)** ROC AUC score, **(B)** Accuracy, **(C)** Recall, and **(D)** Precision. The Y-axis lists classifier names; the X-axis indicates each corresponding performance score. Models evaluated include Extra Trees, XGBoost, Random Forest, LightGBM, CatBoost, Gradient Boosting, K-Nearest Neighbors (KNN), Decision Tree, AdaBoost, Naive Bayes, Quadratic Discriminant Analysis (QDA), Linear Discriminant Analysis (LDA), and Logistic Regression.

**Figure 4:**
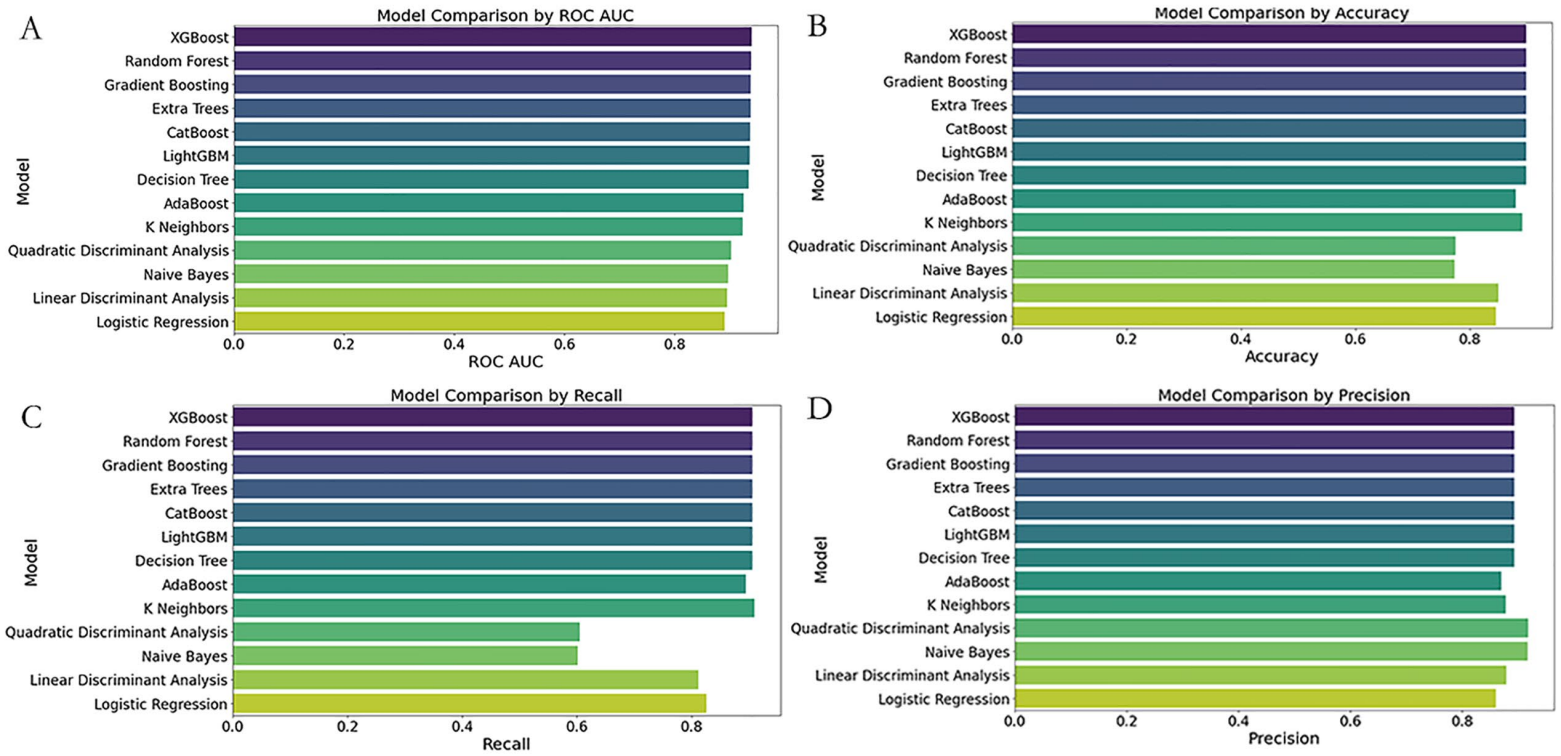
Comparative performance of 13 classifiers using simplified ED predictors. Subfigures **(A–D)** compare the performance of 13 classification models using four evaluation metrics: **(A)** ROC AUC score, **(B)** Accuracy, **(C)** Recall, and **(D)** Precision. The Y-axis lists classifier names; the X-axis indicates each corresponding performance score. Models evaluated include Extra Trees, XGBoost, Random Forest, LightGBM, CatBoost, Gradient Boosting, K-Nearest Neighbors (KNN), Decision Tree, AdaBoost, Naive Bayes, Quadratic Discriminant Analysis (QDA), Linear Discriminant Analysis (LDA), and Logistic Regression.

These results indicate that stacked models are constrained by data sparsity and feature loss in simplified triage scenarios. In contrast, XGBoost retained strong generalizability due to its efficient gradient boosting algorithm. Future work should prioritize optimizing feature engineering to minimize information loss and explore adaptive thresholding or hierarchical ensemble strategies while avoiding overcomplicated architectures that may degrade performance (Figures 3, 4).

#### 3.5.2 Further performance evaluation

##### 3.5.2.1 Prediction performance of the extra trees classifier using full feature set

To ensure optimal model performance and validate its robustness, we employed a two-step strategy. First, we optimized hyperparameters of the Extra Trees Classifier using grid search with cross-validation (GridSearchCV). This involved a comprehensive search over multiple hyperparameter combinations, including the number of trees, maximum depth, and minimum samples required for splitting, with the goal of maximizing the ROC AUC score. The optimal combination of hyperparameters was identified using 10-fold cross-validation on the training dataset, and the model was retrained accordingly.

**Figure 5:**
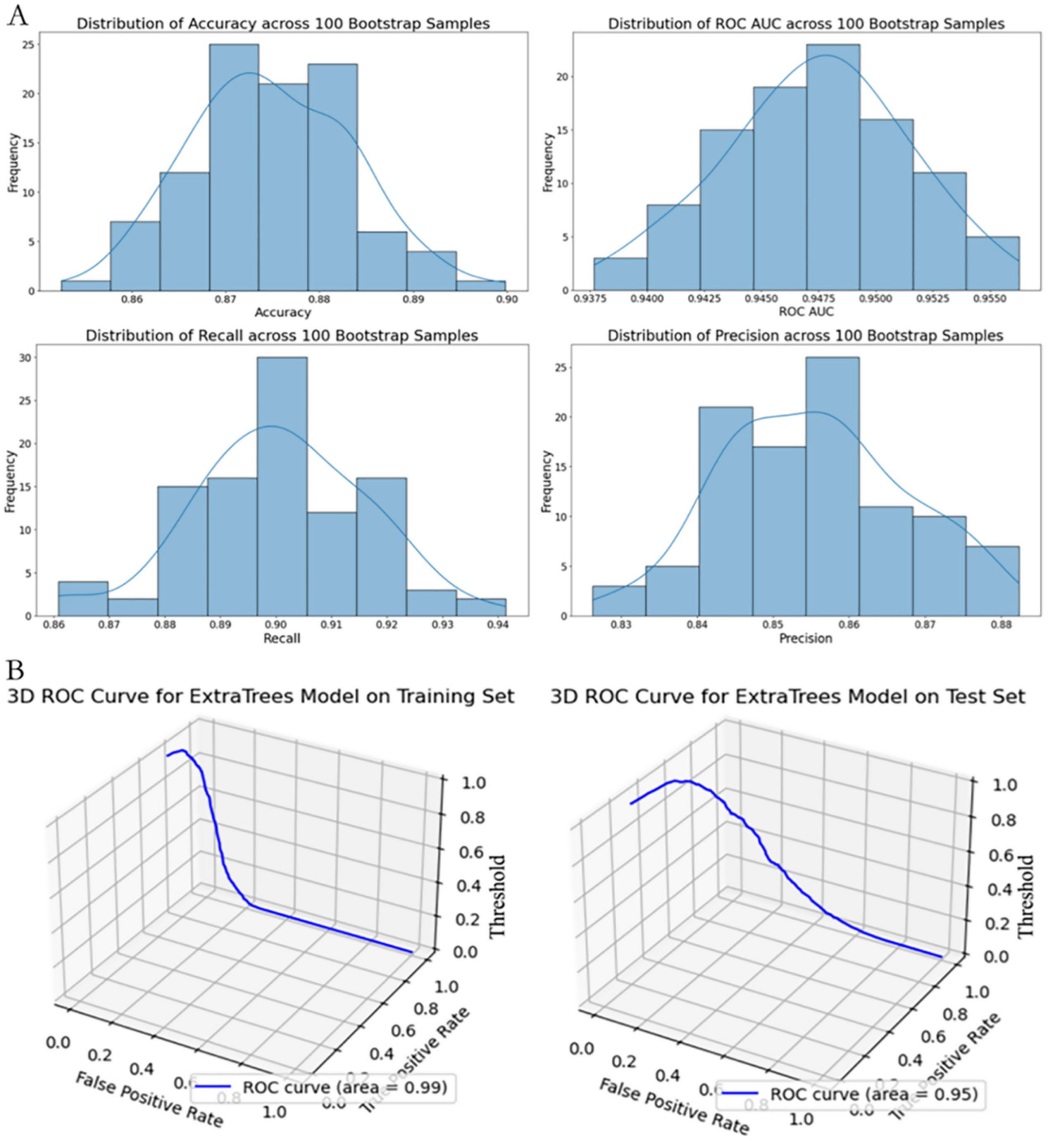
Performance stability and 3D ROC visualization of the extra trees model using full predictor set. **(A)** Distribution of performance metrics (Accuracy, ROC AUC, Recall, Precision) based on 100 bootstrap resamples. Histograms with fitted KDE curves demonstrate the model’s internal stability across evaluation metrics. **(B)** 3D visualization of the ROC curve on the training set (left) and test set (right), plotted with axes representing False Positive Rate (X-axis), True Positive Rate (Y-axis), and classification Threshold (Z-axis).

As shown in Figure 5, subsequently, we conducted 100 rounds of bootstrap resampling. In each iteration, the model was retrained and evaluated on the test set. The performance metrics—including accuracy, ROC AUC, recall, and precision—consistently showed high values with stable distributions (Figure 5). After optimization, the Extra Trees model achieved perfect scores (accuracy, ROC AUC, recall, and precision all equal to 1.0) on the training set. On the test set, it maintained achieved high discrimination in this dataset with an accuracy of 0.889, a ROC AUC of 0.959, a recall of 0.919, and a precision of 0.866.

##### 3.5.2.2 Prediction performance of XGBoost model using simplified ED indicators

Using a reduced set of easily accessible clinical indicators for emergency triage—namely GCS < 15, presence of underlying disease, hypotension, dyspnea, and generalized urticaria—we developed a streamlined XGBoost prediction model. The training dataset was balanced using the ADASYN algorithm and stratified into a 70:30 training-test split (*n* = 659 for training, *n* = 283 for testing).

Hyperparameters were optimized via grid search with three-fold stratified cross-validation, focusing on key parameters such as learning rate (0.01–0.3), maximum tree depth (3–7), and subsample ratio (0.6–1.0), with ROC AUC as the primary evaluation metric.

The tuned XGBoost model achieved high discriminatory performance in the training set (accuracy = 0.9213, ROC AUC = 0.9872, recall = 0.9231, precision = 0.9132). Given the small number of high-risk cases, these results may overestimate real-world applicability and require external validation. On the independent test set, the model demonstrated good generalization with an accuracy of 0.8894 (95% CI, 0.854–0.921), ROC AUC of 0.9397 (95% CI, 0.912–0.962), recall of 0.9058, and precision of 0.8929.

**Figure 6:**
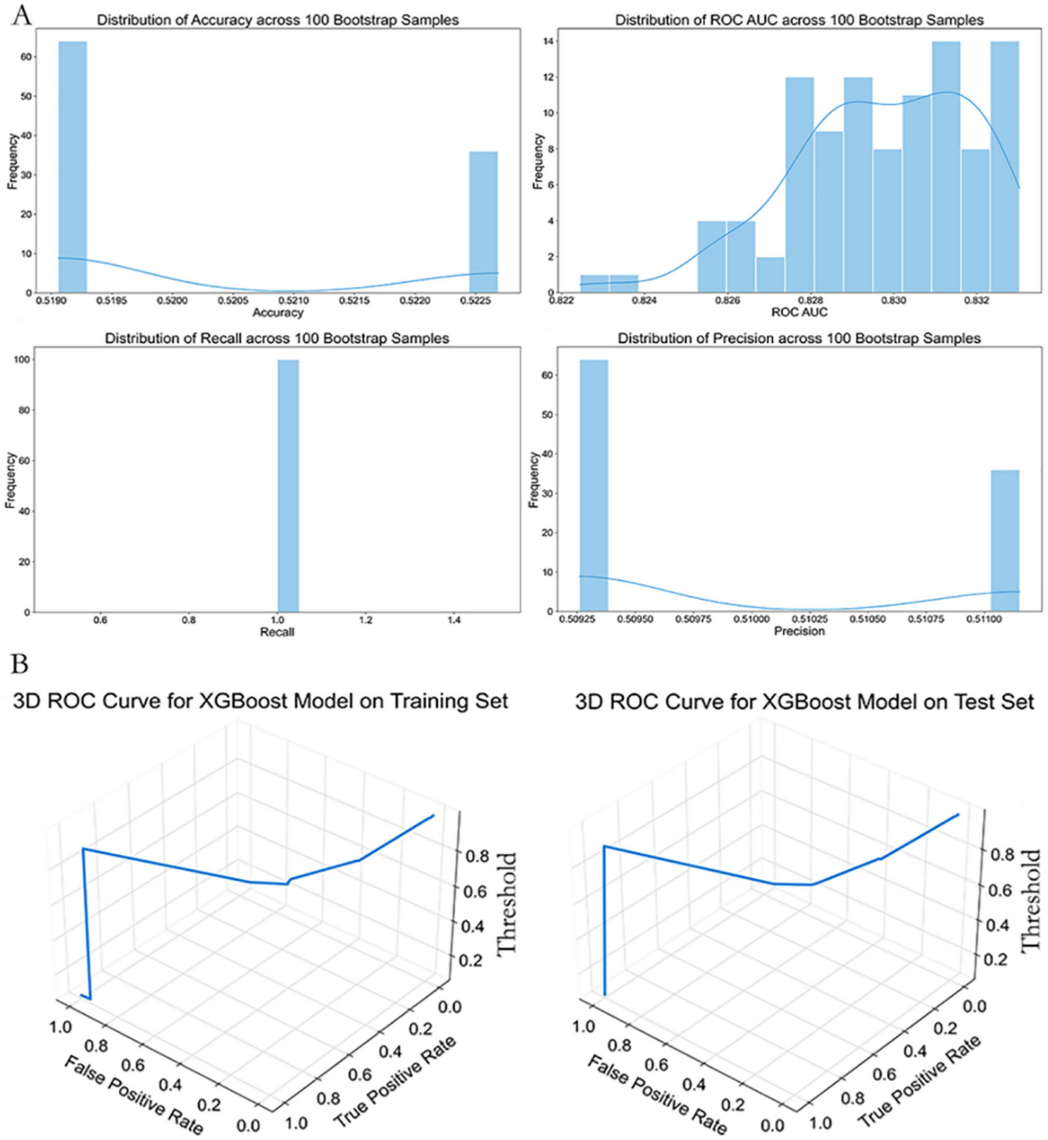
Performance stability and 3D ROC curve of the simplified XGBoost model. **(A)** Distributions of classification metrics (Accuracy, ROC AUC, Recall, Precision) from 100 bootstrap samples based on the simplified XGBoost model. The histograms show variability and reliability of performance estimates across random resampling. **(B)** Three-dimensional ROC curves plotted for the training (left) and test (right) datasets. Axes represent False Positive Rate (X-axis), True Positive Rate (Y-axis), and classification threshold (Z-axis), providing a more intuitive understanding of model behavior under varying threshold values.

As shown in Figure 6, to further assess robustness, we conducted 100 rounds of bootstrap sampling. The test set metrics remained highly stable, with mean accuracy of 0.883 ± 0.021, ROC AUC of 0.933 ± 0.017, recall of 0.901 ± 0.034, and precision of 0.885 ± 0.039 (Figure 6A).

Three-dimensional ROC curve visualizations confirmed desirable threshold responsiveness in both training and test sets (Figure 6B). At a probability threshold of 0.45, the model achieved optimal performance in the test set, with sensitivity of 0.914 and specificity of 0.867.

Notably, although the ROC AUC of the simplified model was approximately 2.2% lower than that of the full-feature Extra Trees model (0.959 vs. 0.9397), it offered significant clinical convenience by reducing the number of required features from seven to five, all of which are readily obtainable at the point of ED triage.

As shown in Table 1, Based on the improved performance following multiple imputation, all primary performance metrics reported hereafter were derived from the MICE-imputed dataset. The Extra Trees and simplified XGBoost classifiers retained strong discrimination (AUC up to 0.9971; Table 1); however, given the limited number of high-risk cases, these values may overestimate real-world performance, underscoring the need for prospective validation in external cohorts. This approach provides greater reproducibility and more realistic variance estimates for clinical application.

For comparison, earlier results based on mean imputation yielded slightly lower performance (e.g., AUC 0.959 for Extra Trees), confirming the robustness and superiority of the MICE-imputed approach.

To evaluate the impact of imputation strategies, model performance using mean-imputed and MICE-imputed datasets was compared (Table 1). Both Extra Trees and simplified XGBoost classifiers exhibited substantial improvements under MICE imputation, particularly in recall and precision, underscoring the robustness and clinical utility of the multiple imputation approach. For comparison, earlier results based on mean imputation yielded slightly lower performance (e.g., AUC 0.959 for Extra Trees), confirming the superiority of the MICE-imputed strategy.

#### 3.5.3 Model interpretation

To enhance the interpretability of the predictive model, we applied SHapley Additive exPlanations (SHAP) to evaluate the feature importance and decision rationale of the Extra Trees model. The results are shown in Figure 5.

**Figure 7:**
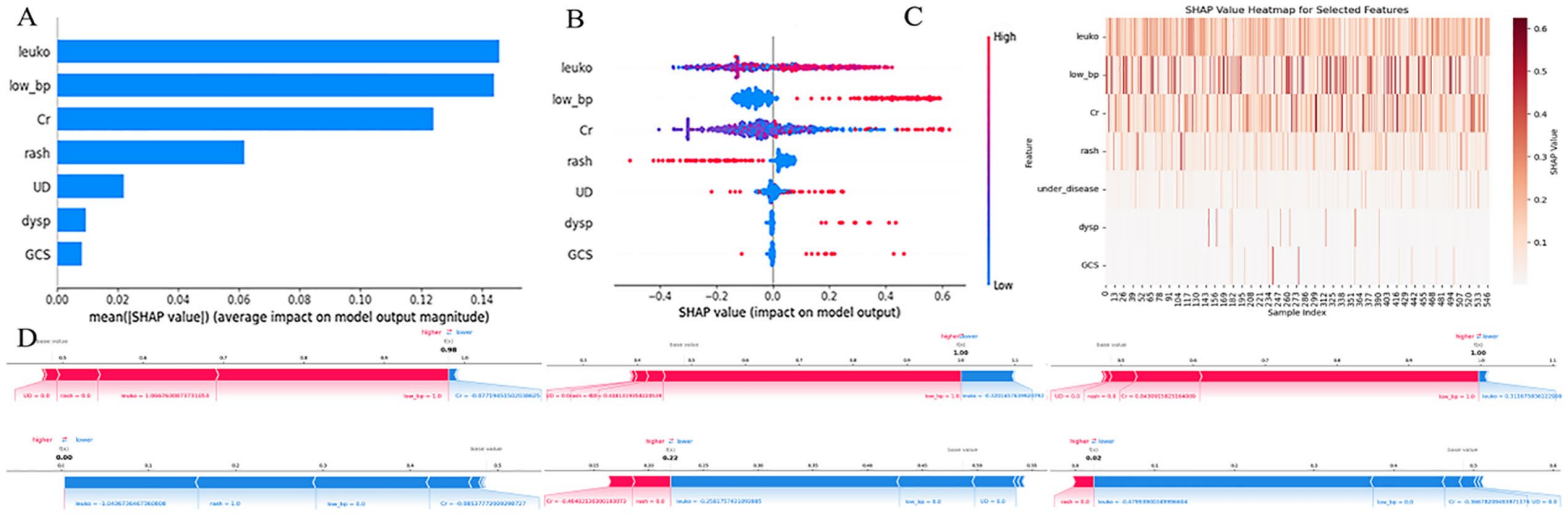
SHAP-based interpretation of the extra trees model. **(A)** Bar plot showing the average absolute SHAP values for each predictor, indicating their relative contributions to model output across all samples. **(B)** Beeswarm plot visualizing SHAP value distributions for each feature. Color represents the feature value (red = high, blue = low). **(C)** SHAP heatmap illustrating the magnitude and direction of each feature’s contribution across all samples. **(D)** SHAP force plots for three individual patients, showing how each feature shifts the prediction toward higher or lower risk. X-axis in most subplots represents SHAP values; Y-axis denotes either features or samples, depending on the plot; color gradients reflect input feature values. leuko, Leukocyte count; low_bp, ow blood pressure; Cr, serum creatinin; UD, underlying disease; dysp, dyspnea; GCS, GCS < 15.

Figure 7A illustrates the mean absolute SHAP values for each feature, representing their average contribution to the model’s predictions. Leukocyte count (leuko), low blood pressure (low_bp), and serum creatinine (Cr) emerged as the most influential predictors. Their mean SHAP values were substantially higher than those of other features, indicating a dominant role in determining model output. In contrast, rash, underlying disease (UD), dyspnea (dysp), and GCS contributed less on average.

Figure 7B (beeswarm plot) displays the distribution of SHAP values for each feature, showing how variations in feature values influenced the model output. For example, higher leukocyte counts were associated with increased SHAP values (i.e., stronger prediction of adverse outcomes), while lower counts reduced the predicted risk. A similar pattern was observed for low_bp and Cr, supporting their positive contribution to high-risk prediction. Figure 7C presents a SHAP heatmap of selected features across individual samples. Notably, leuko and low_bp consistently showed higher SHAP values in most cases, reinforcing their key influence, whereas dysp and GCS<15 had lower contributions in the majority of samples.

Figure 7D provides SHAP force plots for three representative cases, illustrating individual-level explanations of the model’s predictions. In these cases, leuko, low_bp, and Cr exerted strong positive effects, driving the prediction toward a high-risk classification, while features such as rash and UD sometimes had negative contributions, mitigating the predicted risk.

SHAP interpretation of the simplified ED triage model (XGBoost) is shown in Figure 6, where low blood pressure (low_bp), GCS < 15, and underlying disease (UD) were the most impactful predictors. As illustrated in the SHAP beeswarm plot (Figure 7A), high values of low_bp (red dots) were strongly associated with positive SHAP values, suggesting a higher risk of adverse outcome. GCS < 15 also demonstrated a right-skewed SHAP distribution, emphasizing altered consciousness as a critical danger sign. Interestingly, UD displayed a bimodal SHAP distribution, implying its importance varies across patient subgroups.

**Figure 8:**
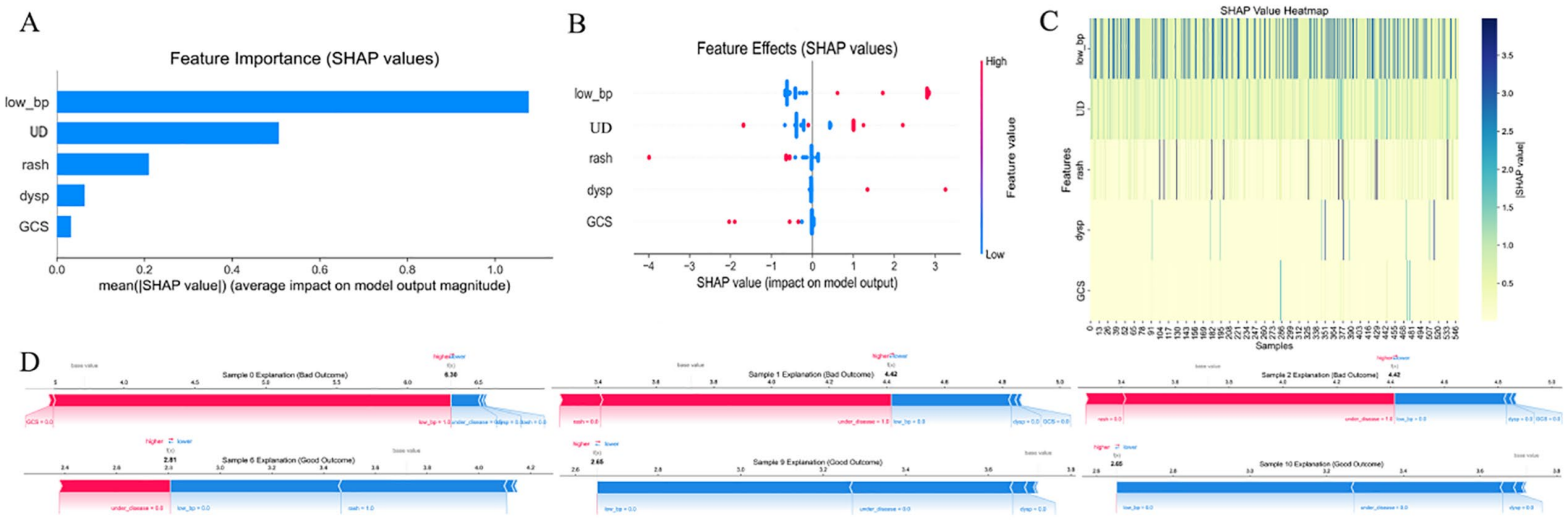
SHAP-based feature interpretation of the simplified XGBoost model. **(A)** Bar plot of mean absolute SHAP values, ranking the features by average impact on model output. **(B)** Beeswarm plot showing the distribution of SHAP values for each predictor, where color denotes feature value (red = high, blue = low). **(C)** Heatmap of SHAP values across samples, visualizing variability and feature dominance. **(D)** SHAP force plots for three patients, showing how individual features push the model prediction toward high- or low-risk outcomes. X-axis in most subplots represents SHAP values; Y-axis denotes either features or samples, depending on the plot; color gradients reflect input feature values. low_bp, Low Blood Pressure; UD, Underlying Disease; dysp, Dyspnea; GCS, GCS < 15. SHAP, SHapley Additive exPlanations.

As shown in Figure 8, Compared with the full model (Figure 1), the importance of dyspnea (dysp) and rash (generalized urticaria) was lower in the simplified model, potentially due to multicollinearity. The SHAP dependence plot (Figure 8B) revealed that co-occurrence of low_bp and GCS < 15 led to a nonlinear surge in predicted risk, indicating a synergistic interaction between these features in influencing model decisions.

Bootstrap validation (Figures 8C, D) demonstrated the model’s robustness, with coefficient of variation for SHAP values across 100 resampling iterations remaining below 15%, confirming the model’s reliability in clinical application scenarios.

#### 3.5.4 Model deployment

Based on the optimized ensemble model, we developed a shareable web-based risk calculator to facilitate real-time clinical decision-making. The tool incorporates seven common clinical variables—underlying disease, Glasgow Coma Scale < 15 (GCS < 15), hypotension (systolic blood pressure <90 mmHg or diastolic <60 mmHg), dyspnea, generalized urticaria, syncope, leukocyte count, and serum creatinine—and enables physicians to dynamically assess the probability and risk level of high-risk hymenopteran sting injury upon patient admission via mobile or desktop web interfaces.

**Figure 9:**
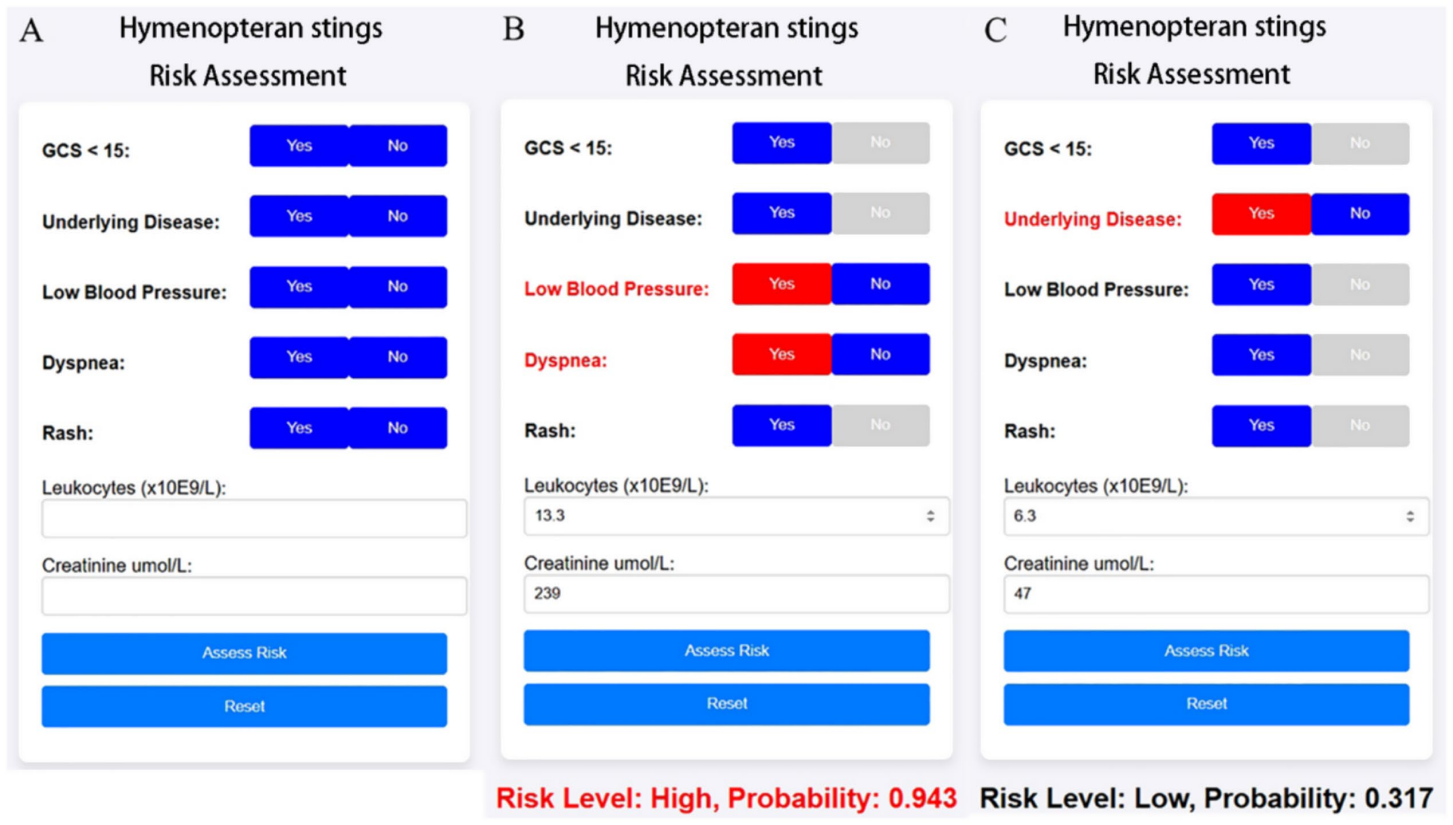
Web-based clinical calculator for risk stratification of hymenopteran sting cases. The calculator dynamically outputs predicted risk level and probability score based on the simplified XGBoost model. It is intended for real-time clinical support and triage optimization in emergency departments. **(A)** Initial interface of the online risk calculator, allowing clinicians to input patient characteristics including symptoms (e.g., dyspnea, rash), vital signs (e.g., blood pressure), GCS score, and laboratory values (leukocyte count, creatinine). **(B)** Example of a high-risk prediction: multiple risk factors are selected, resulting in a predicted risk probability of 0.943. Red highlights indicate contributing risk features. **(C)** Example of a low-risk case: fewer abnormalities are present, leading to a lower probability (0.317).

As shown in Figure 9, As illustrated in Figure 9B, for a 38-year-old male patient with no prior medical history who presented to the hospital 10 h after being stung by a wasp., the calculator estimated a 94.3% probability of high-risk hymenopteran sting injury. This patient had hypotension (systolic BP = 81 mmHg), dyspnea, leukocyte count of 13.3 × 10^9^/L, and serum creatinine of 239 μmol/L, leading to classification into the high-risk group.

In contrast, Figure 9C shows a 58-year-old female patient with a history of hypertension but no impaired consciousness, hypotension, dyspnea, or rash. Her leukocyte count was 6.3 × 10^9^/L and serum creatinine was 47 μmol/L. For this case, the web-based tool predicted a 31.7% probability of high-risk hymenopteran sting injury, categorizing her into the low-risk group.

## 4 Discussion

This study, based on multicenter clinical data from 942 patients with hymenopteran stings, systematically analyzed epidemiological patterns, identified key variables associated with adverse outcomes, developed high-performance machine learning prediction models, and deployed a simplified online risk assessment tool. Together, these efforts offer both theoretical foundations and practical strategies for early recognition and precise intervention in patients with hymenopteran sting injuries. To our knowledge, this is among the first applications of SHAP-based interpretability to Hymenoptera envenomation risk prediction.

### 4.1 Epidemiological and clinical risk features

Our findings indicate that hymenopteran stings occur predominantly in summer and autumn, with over two-thirds of cases reported in the third quarter—consistent with the seasonal activity peak of social Hymenoptera (wasps, hornets, bees and bumblebees), when colony sizes and foraging activity are greatest (12). The head and neck were the most commonly affected anatomical sites. A higher proportion of cases occurred in males and older adults, suggesting that outdoor exposure and physiological vulnerability play critical roles in injury progression. Notably, underlying comorbidities were more prevalent among patients with poor outcomes, reinforcing the notion of wasp envenomation as an “exogenous triggering factor” for systemic injury.

Analysis of high-risk clinical features revealed that hypotension, altered mental status, dyspnea, elevated leukocyte counts, and abnormal creatinine levels were significantly associated with adverse outcomes. These indicators align with known pathophysiological mechanisms of wasp venom. Specifically, phospholipase A2 and hyaluronidase in venom can damage endothelial cells and trigger inflammatory cascades, leading to increased vascular permeability, rhabdomyolysis, and acute kidney injury (13). As a result, leukocyte and creatinine levels often rise markedly. Furthermore, hypotension and impaired consciousness—indicative of inadequate organ perfusion and systemic inflammation—are of high clinical relevance in acute severity assessment. Although the predictive importance of “rash” was relatively limited, this variable was retained in the simplified model to enhance clinical interpretability and facilitate ease of application in bedside settings.

### 4.2 Machine learning model performance and clinical AI applications

In comparing 13 mainstream machine learning algorithms, the Extra Trees model exhibited the best performance on the test set (accuracy = 0.889, AUC = 0.959), outperforming logistic regression, naïve Bayes, and other models. Extra Trees excelled in capturing complex feature interactions and nonlinear decision boundaries. Its interpretability, enhanced through SHAP (SHapley Additive exPlanations), revealed that leukocyte count, hypotension, and creatinine were the top three contributors to risk predictions—offering clinicians transparent insight into model rationale.

To enhance clinical applicability, we also developed a simplified model based on five routinely available emergency indicators. Although the AUC slightly declined to 0.937, the model’s ease of use and rapid data acquisition make it suitable for prehospital triage and community-level emergency care. With individual SHAP value visualizations, this streamlined model enables personalized risk factor analysis, supporting frontline clinicians in formulating dynamic intervention strategies. The deployment of this model as a web-based tool represents a significant advancement in point-of-care clinical decision support systems for hymenopteran envenomation (2, 14).

The application of machine learning to hymenopteran sting risk prediction fills a critical gap in emergency toxicology. Unlike traditional scoring systems that rely on fixed weights and linear relationships, our ensemble approach can capture complex interactions between clinical variables that may not be apparent to human observers (15). This is particularly relevant in envenomation scenarios where the interaction between patient comorbidities, venom load, and systemic response can lead to unpredictable clinical trajectories.

MICE outperformed mean imputation, supporting model robustness. As detailed in Table 1, both the Extra Trees and simplified XGBoost models trained on MICE-imputed datasets significantly outperformed those using mean substitution, particularly in recall and precision. These findings validate the importance of preserving feature variance and minimizing bias in emergency care data, and justify the adoption of MICE-based results as the primary analysis. Given the small number of high-risk cases (*n* = 23), these high AUC values may overestimate real-world performance. Future prospective validation in external cohorts is necessary to confirm the generalizability of the model. However, the high AUC values observed should be interpreted cautiously due to the small number of high-risk cases.

### 4.3 Comparison with traditional risk scores

Compared with traditional scoring systems such as SOFA, qSOFA, and NEWS—which are widely used in sepsis and acute care evaluation—our SHAP-enhanced Extra Trees and simplified XGBoost models demonstrated substantially higher predictive performance in hymenopteran sting cases (AUC up to 0.959) (10, 16). These conventional tools rely on fixed thresholds and a limited number of clinical parameters, which may reduce sensitivity and specificity in this specific clinical context. In contrast, our models dynamically integrate a broader range of clinical and laboratory features, are capable of generating individualized explanations of risk contribution via SHAP, and allow rapid deployment in emergency settings through a streamlined feature set and an online calculator interface (17). This combination of precision, transparency, and operational feasibility offers distinct advantages over SOFA, qSOFA, and NEWS in guiding early, targeted intervention for high-risk hymenopteran sting patients (7, 10).

### 4.4 Limitations

This study has several limitations. First, the dataset was drawn from five hospitals in Hainan Province. While it offers regional representativeness, the geographical and demographic diversity remains limited. Second, the absence of information regarding bee species, venom dosage, and treatment delay restricted our ability to explore dose–response relationships. Third, although the model performed consistently on internal test data, external validation in other regions and prospective cohorts is necessary to ensure generalizability and robustness.

Furthermore, the model is a clinical decision support tool requiring physician oversight to mitigate ethical and safety risks from algorithmic misclassification. In cases of missing input features or low model confidence, clinicians should default to their professional expertise.

While ADASYN oversampling effectively mitigated class imbalance in the training data, synthetic samples cannot fully replace real-world cases. Therefore, external validation in independent cohorts is necessary to confirm model generalizability and avoid potential overfitting.

Public access to the web-based risk calculator is temporarily suspended due to infrastructure maintenance. Full source code and deployment instructions are provided in Supplementary material S1 for reproducibility. Public URL access is expected to resume within 3 months post-publication.

## 5 Conclusion

In conclusion, we developed and validated an interpretable, streamlined, and readily deployable model for early identification of patients at high risk of severe outcomes following hymenopteran stings, leveraging real-world multicenter data. This model fills a critical gap in risk stratification for hymenopteran envenomation and demonstrates clear potential for improving emergency triage and public health response. Future prospective validation in diverse populations is warranted to establish its broader generalizability and clinical impact.

## Data Availability

The raw data supporting the conclusions of this article will be made available by the authors, without undue reservation.

## Supplementary material

SUPPLEMENTARY FIGURE S1 Youden index-based threshold analysis for serum creatinine.

SUPPLEMENTARY FIGURE S2 Youden index-based threshold analysis for leukocyte count.
