# Supplementary figures and images for: Machine learning enables early risk stratification of hymenopteran stings: evidence from a tropical multicenter cohort
Source: Front Public Health. 2025 Oct 28;13:1664606. doi: 10.3389/fpubh.2025.1664606 (PMC12602473; doi:10.3389/fpubh.2025.1664606)

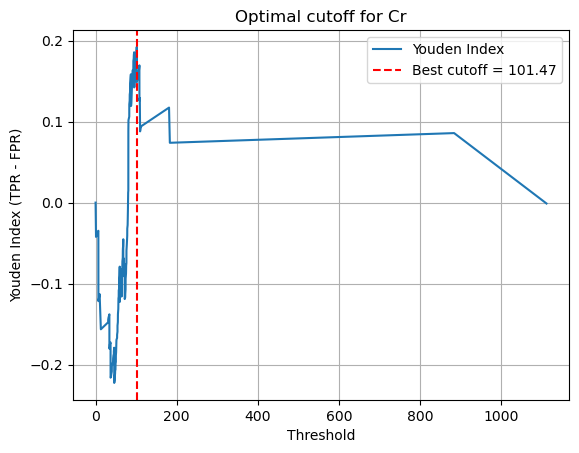

Supplement: SUPPLEMENTARY FIGURE S1 — Youden index-based threshold analysis for serum creatinine. [file Image_1.PNG]

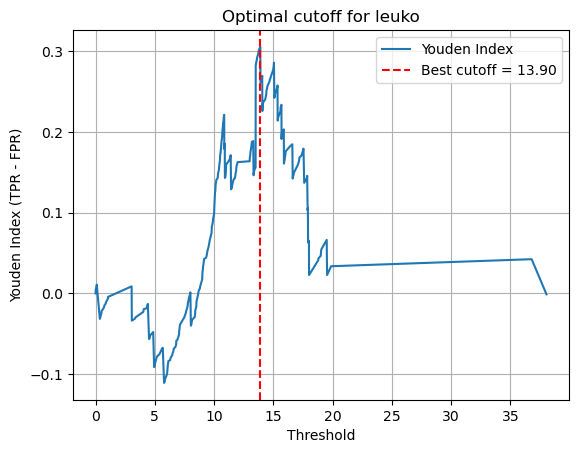

Supplement: SUPPLEMENTARY FIGURE S2 — Youden index-based threshold analysis for leukocyte count. [file Image_2.PNG]
