# Supplementary material for: Machine learning enables early risk stratification of hymenopteran stings: evidence from a tropical multicenter cohort
Source: Front Public Health. 2025 Oct 28;13:1664606. doi: 10.3389/fpubh.2025.1664606 (PMC12602473; doi:10.3389/fpubh.2025.1664606)
Supplement: Supplementary file 3 [file Supplementary_file_1.docx]

# Supplementary Material S1: Web-based Risk Calculator Source Code

This supplementary file contains the complete source code for the bee sting risk calculator, including backend (Flask) and frontend (HTML+JavaScript) components. The tool can be deployed locally or on an institutional server. Please ensure Python 3.7+, Flask, and required packages are installed.

## 1. Backend: Flask Application (app.py)

from flask import Flask, request, jsonify, render_template
import pickle
import numpy as np

app = Flask(__name__)

with open('naive_bayes_model.pkl', 'rb') as file:
 model = pickle.load(file)

@app.route('/')
def home():
 return render_template('index.html')

@app.route('/predict', methods=['POST'])
def predict():
 data = request.json
 features = np.array(data['features']).reshape(1, -1)
 prediction = model.predict_proba(features)[0][1]
 risk_level = 'High' if prediction > 0.5 else 'Low'
 return jsonify({'probability': prediction, 'risk_level': risk_level})

if __name__ == '__main__':
 app.run(debug=False, host='0.0.0.0', port=80)

## 2. Frontend: HTML Page (templates/index.html)

<!DOCTYPE html>

<html lang="en">

<head>

<meta charset="UTF-8">

<title>Bee Sting Risk Calculator</title>

<script src="https://code.jquery.com/jquery-3.6.0.min.js"></script>

<style>

body {

font-family: Arial, sans-serif;

padding: 20px;

}

label {

display: block;

margin-bottom: 8px;

font-weight: bold;

}

input[type="text"] {

padding: 10px;

border-radius: 10px;

border: 1px solid #ccc;

box-shadow: 2px 2px 4px rgba(0,0,0,0.2);

width: 220px;

margin-bottom: 20px;

}

.toggle-group {

display: flex;

gap: 12px;

margin-bottom: 20px;

}

.toggle-btn {

padding: 10px 24px;

border-radius: 8px;

background-color: #ccc;

color: white;

font-weight: bold;

cursor: pointer;

box-shadow: 2px 2px 5px rgba(0,0,0,0.2);

}

.selected-yes {

background-color: #e60000 !important;

}

.selected-no {

background-color: #005ce6 !important;

}

.flashing {

animation: flash 0.4s ease-in-out 3;

}

@keyframes flash {

0%, 100% { background-color: #ccc; }

50% { background-color: white; }

}

button {

padding: 10px 28px;

border: none;

background-color: #0073e6;

color: white;

font-size: 16px;

border-radius: 6px;

cursor: pointer;

margin-right: 10px;

}

</style>

</head>

<body>

<h2>Bee Sting Risk Calculator</h2>

<form id="risk-form" onsubmit="return false;">

<label for="leukocyte">Leukocyte (10⁹/L)</label>

<input type="text" id="leukocyte">

<label for="creatinine">Creatinine (μmol/L)</label>

<input type="text" id="creatinine">

<label>Low Blood Pressure:</label>

<div class="toggle-group" data-id="low_bp">

<div class="toggle-btn" data-value="1">YES</div>

<div class="toggle-btn" data-value="0">NO</div>

</div>

<label>Underlying Disease:</label>

<div class="toggle-group" data-id="under_disease">

<div class="toggle-btn" data-value="1">YES</div>

<div class="toggle-btn" data-value="0">NO</div>

</div>

<button onclick="validateForm()">Assess Risk</button>

<button type="button" onclick="resetForm()">Reset</button>

</form>

<script>

let selected = {};

$('.toggle-btn').click(function () {

const group = $(this).closest('.toggle-group');

const id = group.data('id');

const val = $(this).data('value');

selected[id] = val;

group.find('.toggle-btn').removeClass('selected-yes selected-no');

if (val === 1) {

$(this).addClass('selected-yes');

} else {

$(this).addClass('selected-no');

}

});

function validateForm() {

let valid = true;

["#leukocyte", "#creatinine"].forEach(id => {

if (!$(id).val()) {

flash($(id));

valid = false;

}

});

$(".toggle-group").each(function () {

const id = $(this).data('id');

if (!(id in selected)) {

flash($(this).children());

valid = false;

}

});

}

function flash(el) {

el.addClass("flashing");

setTimeout(() => el.removeClass("flashing"), 1200);

}

function resetForm() {

$("#leukocyte, #creatinine").val("");

$(".toggle-btn").removeClass("selected-yes selected-no");

selected = {};

}

</script>

</body>

</html>
